# A Multicenter, Randomized, Double-Blind, Phase 2 Study of the Efficacy and Safety of Plazomicin Compared with Levofloxacin in the Treatment of Complicated Urinary Tract Infection and Acute Pyelonephritis

**DOI:** 10.1128/AAC.01989-17

**Published:** 2018-03-27

**Authors:** Lynn E. Connolly, Valerie Riddle, Deborah Cebrik, Eliana S. Armstrong, Loren G. Miller

**Affiliations:** a Achaogen, Inc., South San Francisco, California, USA; b BioPharmAdvisors LLC, Parrish, Florida, USA; c David Geffen School of Medicine, University of California, Los Angeles, Los Angeles BioMedical Research at Harbor-UCLA Medical Center, Torrance, California, USA

**Keywords:** aminoglycosides, antibacterial therapy, clinical trials, complicated urinary tract infection, plazomicin, pyelonephritis

## Abstract

Increasing antimicrobial resistance among uropathogens limits treatment options for patients with complicated urinary tract infection (cUTI). Plazomicin, a new aminoglycoside, has *in vitro* activity against multidrug-resistant Enterobacteriaceae, including isolates resistant to currently available aminoglycosides, as well as extended-spectrum β-lactamase-producing and carbapenem-resistant Enterobacteriaceae. We evaluated the efficacy and safety of plazomicin in a double-blind, comparator-controlled, phase 2 study in adults with cUTI or acute pyelonephritis. Patients were randomized 1:1:1 to receive intravenous plazomicin (10 or 15 mg/kg of body weight) or intravenous levofloxacin (750 mg) once daily for 5 days. Coprimary efficacy endpoints were microbiological eradication at the test of cure (TOC; 5 to 12 days after the last dose) in the modified intent-to-treat (MITT) and microbiologically evaluable (ME) populations. Overall, 145 patients were randomized to treatment. In the groups receiving plazomicin at 10 mg/kg, plazomicin at 15 mg/kg, and levofloxacin, microbiological eradication rates were, respectively, 50.0% (6 patients with microbiological eradication at TOC/12 patients treated [95% confidence interval {CI}, 21.1 to 78.9%]), 60.8% (31/51 [95% CI, 46.1 to 74.2%]), and 58.6% (17/29 [95% CI, 38.9 to 76.5%]) in the MITT population and 85.7% (6/7 [95% CI, 42.1 to 99.6%]), 88.6% (31/35 [95% CI, 73.3 to 96.8%]), and 81.0% (17/21 [95% CI, 58.1 to 94.6%]) in the ME population. In the MITT population, 66.7% (95% CI, 34.9 to 90.1%), 70.6% (95% CI, 56.2 to 82.5%), and 65.5% (95% CI, 45.7 to 82.1%) of the patients in the three groups, respectively, were assessed by the investigator to be clinically cured at TOC. Adverse events were reported in 31.8%, 35.1%, and 47.7% of the patients in the three groups, respectively. Serum creatinine values were generally stable over the course of the study. No plazomicin-treated patients with evaluable audiometry data had postbaseline sensorineural, conductive, or mixed hearing loss. In summary, plazomicin demonstrated microbiological and clinical success and an overall safety profile supportive of further clinical development. (This study has been registered at ClinicalTrials.gov under identifier NCT01096849.)

## INTRODUCTION

Bacterial urinary tract infections (UTIs) are common in both community and hospital settings and pose a substantial burden to patients and health care systems (1, 2). Complicated UTIs (cUTIs) and acute pyelonephritis (AP) can be challenging to treat and often require parenteral therapy (3).

The majority of cUTIs are caused by Enterobacteriaceae, most commonly, Escherichia coli (4). Treatment of these infections is increasingly challenging due to the global rise of antibacterial resistance (4–9). Multidrug-resistant (MDR) Enterobacteriaceae, including extended-spectrum β-lactamase (ESBL)- and carbapenemase-producing organisms, limit treatment options for patients with cUTIs (7, 8, 10–13). Antibiotic-resistant Enterobacteriaceae causing cUTIs are associated with lengthy hospital stays, increased health care expenditures, and elevated rates of mortality (1, 14–19). New antibiotics for the treatment of infections due to MDR Enterobacteriaceae are urgently needed (20, 21).

Aminoglycosides are a well-established class of antibiotics that are especially useful in the treatment of serious infections caused by Gram-negative bacteria due to their rapid, concentration-dependent bactericidal action and ability to act synergistically with other antibiotics (22). Although the use of aminoglycosides has declined over the years due to concerns about toxicity, they have recently reemerged as a practical approach to treating patients with cUTIs caused by MDR Gram-negative bacteria (23–25). Ideally, aminoglycosides are dosed once daily, a treatment strategy that has been shown to reduce toxicity while maintaining efficacy compared with multiple daily doses (26–29). However, data from large, prospective randomized controlled trials evaluating the safety and efficacy of once-daily aminoglycosides in cUTIs are limited (30).

Plazomicin is a new aminoglycoside derived from sisomicin with structural modifications that protect it from aminoglycoside-modifying enzymes, which are the most common mechanism of resistance to older aminoglycosides (e.g., amikacin, gentamicin, tobramycin) in Enterobacteriaceae (31). *In vitro* studies have shown that plazomicin is rapidly bactericidal and has potent activity against MDR Enterobacteriaceae, including aminoglycoside-resistant (31–33), ESBL-producing (34–36), and carbapenem-resistant (37–40) Enterobacteriaceae.

This phase 2 study evaluated the efficacy and safety of once-daily plazomicin versus once-daily levofloxacin for the treatment of adult patients with cUTIs or AP.

(Part of this research was previously presented at the 52nd Interscience Conference on Antimicrobial Agents and Chemotherapy, 9 to 12 September 2012, San Francisco, CA [41], and the 25th Annual European Congress of Clinical Microbiology and Infectious Diseases, 25 to 28 April 2015, Copenhagen, Denmark.)

## RESULTS

### Patient disposition and baseline characteristics.

**FIG 1:**
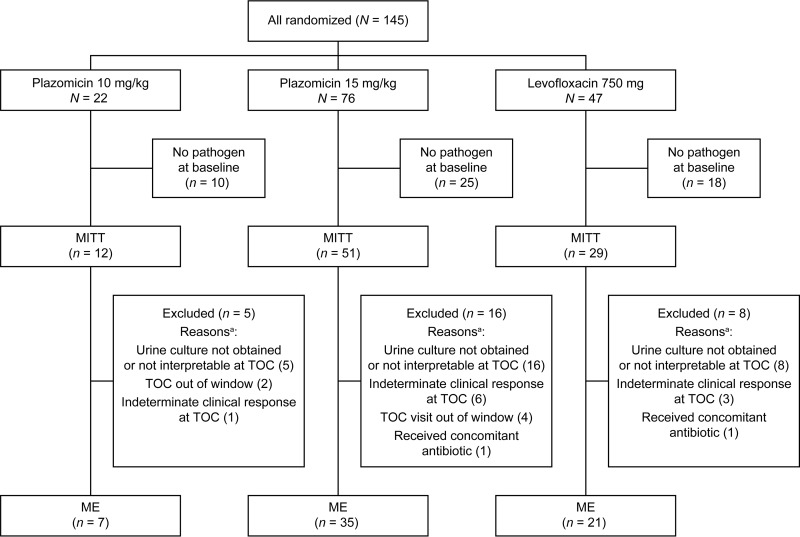
Patient disposition and analysis populations. ^a^, patients could have more than one reason for exclusion.

A total of 145 patients were randomized to treatment (22, 76, and 47 to treatment with plazomicin at 10 mg/kg, plazomicin at 15 mg/kg, and levofloxacin, respectively); 125 (86.2%) completed the study; 5 did not receive study drug. Of the randomized patients, 92 (63.4%) qualified for inclusion in the modified intent-to-treat (MITT) population and 63 (43.4%) qualified for inclusion in the microbiologically evaluable (ME) population (Fig. 1). The safety population included 140 patients who received at least one dose of study drug.

Twenty patients were prematurely withdrawn from the study for the following reasons: consent was withdrawn (*n* = 7), loss to follow-up (*n* = 5), administrative issues (*n* = 3), a lack of efficacy (*n* = 2), adverse events (AEs) (*n* = 2), and investigator decision (*n* = 1). The two patients who were prematurely withdrawn from the study due to a lack of efficacy (both of whom were in the group receiving plazomicin at 15 mg/kg) were included in the safety and MITT populations but not the ME population; both were counted as clinical failures at the test of cure (TOC), at which point neither had an interpretable urine culture result. The two patients who were prematurely withdrawn from the study due to AEs (one in the levofloxacin group, who required additional antibiotics for bacteremia, and one in the group receiving plazomicin at 15 mg/kg) were included in the safety population; both patients were clinical failures at TOC.

The demographic and baseline characteristics for the MITT population were similar across treatment groups (Table 1). The patients had a mean age of 42.4 years (range, 18 to 82 years), 83.7% were women, and 48.9% had a diagnosis of AP.

**TABLE 1:** Patient baseline characteristics (MITT population)

Characteristic	Values for patients receiving:		
	Plazomicin at 10 mg/kg (*n* = 12)	Plazomicin at 15 mg/kg (*n* = 51)	Levofloxacin at 750 mg (*n* = 29)
Mean ± SD age (yr)	41.5 ± 20.02	39.5 ± 15.2	47.9 ± 15.1
No. (%) of female patients	10 (83.3)	42 (82.4)	25 (86.2)
No. (%) of patients from the following region:			
North America	4 (33.3)	27 (52.9)	15 (51.7)
India	5 (41.7)	12 (23.5)	6 (20.7)
Latin America	3 (25.0)	12 (23.5)	8 (27.6)
Mean ± SD body wt (kg)	66.4 ± 15.9	68.6 ± 14.5	72.4 ± 13.9
No. (%) of patients with ≥1 prior/ongoing medical disorder	11 (91.7)	44 (86.3)	20 (69.0)
No. (%) of patients with the following primary diagnosis:			
AP	4 (33.3)	24 (47.1)	17 (58.6)
cUTI	8 (66.7)	27 (52.9)	12 (41.4)
No. of patients with cUTI with indwelling catheter/total no. of patients with cUTI (%)	1/8 (12.5)	4/27 (14.8)	1/12 (8.3)
Mean ± SD estimated CL_CR_ (ml/min)^*a*^	109.1 ± 27.8	115.4 ± 54.3	108.3 ± 39.4

a CL_CR_ was estimated for the safety population using the Cockcroft-Gault formula, as follows: CL_CR_ = {([140 − age] × weight)/(72 × serum creatinine concentration)} × 0.85 (if female), where weight is in kilograms and the serum creatinine concentration is in milligrams per deciliter.

### Pathogens at baseline.

In the MITT population, the most commonly isolated organisms were Enterobacteriaceae (*n* = 84), of which 69 were E. coli and 7 were Klebsiella pneumoniae. Only one patient (randomized to the group receiving plazomicin at 15 mg/kg) had more than one Gram-negative organism (Morganella morganii and Proteus mirabilis), quantified at ≥10^5^ CFU/ml at the baseline. Gram-positive bacteria were isolated from six patients.

Among 68 Enterobacteriaceae isolates recovered at the baseline for which MIC data were available, plazomicin MICs ranged from ≤0.12 to 8 μg/ml and levofloxacin MICs ranged from ≤0.12 to >4 μg/ml. Of the two Gram-negative bacterial isolates with plazomicin MICs of >4 μg/ml, one was E. coli and the other was Pseudomonas aeruginosa. Baseline susceptibility testing of Enterobacteriaceae using Clinical and Laboratory Standards Institute (CLSI) (42) and European Committee on Antimicrobial Susceptibility Testing (EUCAST) (43) criteria showed that 27.9% (*n* = 19), including 6 in the levofloxacin treatment group, were resistant to levofloxacin (MIC > 4 μg/ml); 17.6% (*n* = 12) were resistant to ceftazidime by both CLSI and EUCAST criteria; 39.7% (*n* = 27) were resistant to trimethoprim-sulfamethoxazole by CLSI criteria and nonsusceptible per EUCAST criteria; 14.7% (*n* = 10) were resistant to gentamicin by CLSI criteria (MIC ≥ 16 μg/ml) and 17.6% (*n* = 12) were nonsusceptible to gentamicin by EUCAST criteria (MIC ≥ 4 μg/ml); and although none of the isolates were resistant to amikacin by CLSI criteria (MIC ≥ 64 μg/ml), 2 were nonsusceptible by EUCAST criteria (MIC ≥ 16 μg/ml). Overall, 16.2% (*n* = 11) of these Enterobacteriaceae were classified as MDR, defined as nonsusceptible to at least one agent in three or more antimicrobial categories (44).

### Microbiological outcomes.

The microbiological eradication rates at TOC were similar across treatment groups for both the MITT and ME populations (Table 2). The treatment differences between the groups receiving plazomicin at 15 mg/kg and levofloxacin were 2.2% (95% confidence interval [CI], −22.9 to 27.2%) and 7.6% (95% CI, −16.0 to 31.3%) in the MITT and ME populations, respectively. For patients in the ME population, microbiological recurrence at long-term follow-up (LFU) occurred in 6.5% (2/31) and 23.5% (4/17) of the patients in the groups receiving treatment with plazomicin at 15 mg/kg and levofloxacin, respectively.

**TABLE 2:** Microbiological outcome at TOC (primary efficacy endpoint)^*a*^

Population	Treatment	No. of patients	No. (%) of patients with eradication	95% CI for eradication (%)	No. (%) of patients with noneradication	No. (%) of patients with indeterminate outcome^*b*^
MITT	Plazomicin at 10 mg/kg	12	6 (50.0)	21.1–78.9	1 (8.3)	5 (41.7)
	Plazomicin at 15 mg/kg	51	31 (60.8)	46.1–74.2	5 (9.8)	15 (29.4)
	Levofloxacin at 750 mg	29	17 (58.6)	38.9–76.5	4 (13.8)	8 (27.6)
ME	Plazomicin at 10 mg/kg	7	6 (85.7)	42.1–99.6	1 (14.3)	–
	Plazomicin at 15 mg/kg	35	31 (88.6)	73.3–96.8	4 (11.4)	–
	Levofloxacin at 750 mg	21	17 (81.0)	58.1–94.6	4 (19.0)	–

a The difference in microbiological eradication rates between the group receiving plazomicin at 15 mg/kg and the group receiving levofloxacin was 2.2% (95% CI, −22.9 to 27.2%) for the MITT population and 7.6% (95% CI, −16.0 to 31.3%) for the ME population. The 95% CI for the difference was based on a normal approximation with a continuity correction.

b –, patients with missing or indeterminate outcome data were excluded from the ME population.

The microbiological eradication rates by primary diagnosis (AP or cUTIs) and baseline pathogen were generally similar in each treatment group (Table 3). In patients with E. coli at the baseline, 88.0% who received plazomicin at 15 mg/kg and 75.0% who received levofloxacin had a favorable microbiological response at TOC. Although the numbers are small, the data suggest a trend toward lower microbiological eradication rates among patients with baseline pathogens with higher MICs for both plazomicin and levofloxacin (Table 3). Microbiological eradication in the group receiving plazomicin at 15 mg/kg was achieved in 93.1% (27/29) of the patients whose baseline urinary pathogens had a plazomicin MIC of ≤4 μg/ml, including two Enterobacteriaceae isolates with an MIC of 4 μg/ml, and in 66.7% (2/3) of the patients whose pathogens had a plazomicin MIC of >4 μg/ml. Microbiological eradication in the group receiving levofloxacin was achieved in 93.8% (15/16) of patients with levofloxacin-susceptible baseline urinary pathogens (MIC ≤ 4 μg/ml) and 33% (1/3) of patients with levofloxacin-resistant baseline urinary pathogens (MIC > 4 μg/ml). Both patients whose isolates were nonsusceptible to amikacin per EUCAST criteria (one in the plazomicin group, one in the levofloxacin group) had microbiological eradication at TOC.

**TABLE 3:** Microbiological eradication at TOC according to primary diagnosis and baseline pathogen (ME population)

Subgroup	Values for patients receiving:		
	Plazomicin at 10 mg/kg (*n* = 7)	Plazomicin at 15 mg/kg (*n* = 35)	Levofloxacin at 750 mg (*n* = 21)
No. of patients with eradication at TOC/no. of patients with the following primary diagnosis (%):			
AP	2/2 (100)	16/18 (88.9)	12/15 (80.0)
95% CI	15.8–100	65.3–98.6	51.9–95.7
cUTI	4/5 (80.0)	15/17 (88.2)	5/6 (83.3)
95% CI	28.4–99.5	63.6–98.5	35.9–99.6
No. of patients with eradication at TOC/no. of patients infected with the following pathogen at baseline (%):			
Gram-positive bacteria^*a*^	1/1 (100.0)	3/3 (100.0)	1/1 (100.0)
Gram-negative bacteria	5/6 (83.3)	28/32 (87.5)	16/20 (80.0)
E. coli	3/4 (75.0)	22/25 (88.0)	12/16 (75.0)
K. pneumoniae	1/1 (100.0)	2/2 (100.0)	1/1 (100.0)
Other Enterobacteriaceae^*b*^	1/1 (100.0)	4/4 (100.0)	3/3 (100.0)
P. aeruginosa	0/0 (0.0)	1/2 (50.0)	0/0 (0.0)
No. of patients with eradication at TOC/no. of patients whose pathogen had the following MIC at baseline (%):			
Plazomicin MIC ≤ 4 μg/ml	5/6 (83.3)	27/29 (93.1)	16/19 (84.2)
Plazomicin MIC > 4 μg/ml	0/0 (0.0)	2/3 (66.7)	1/1 (100.0)
Levofloxacin MIC ≤ 4 μg/ml	4/4 (100.0)	23/24 (95.8)	15/16 (93.8)
Levofloxacin MIC > 4 μg/ml	1/2 (50.0)	5/7 (71.4)	1/3 (33.3)

a Gram-positive bacteria included methicillin-susceptible Staphylococcus aureus (*n* = 1), Enterococcus faecalis (*n* = 3), and Staphylococcus saprophyticus (*n* = 1).

b Other Enterobacteriaceae included Citrobacter freundii (*n* = 1), Enterobacter aerogenes (*n* = 1), M. morganii (*n* = 2), and P. mirabilis (*n* = 4).

### Clinical outcomes.

Most patients in both treatment groups achieved clinical cure at TOC (Table 4). The median time to clinical cure was 5 days for patients in all three treatment groups (interquartile ranges, 5 to 14 days for the group receiving plazomicin at 10 mg/kg, 5 to 6 days for the group receiving plazomicin at 15 mg/kg, and 5 to 12 days for the group receiving levofloxacin). For patients in the ME population, clinical relapse at LFU occurred in 14.3% (4/28) and 6.3% (1/16) of patients in the groups receiving plazomicin at 15 mg/kg and levofloxacin, respectively.

**TABLE 4:** Clinical outcome at TOC

Population	Treatment	No. of patients	No. (%) of patients cured	95% CI for cure (%)	No. (%) of patients with treatment failure	No. (%) of patients with indeterminate outcome^*a*^
MITT	Plazomicin at 10 mg/kg	12	8 (66.7)	34.9–90.1	3 (25.0)	1 (8.3)
	Plazomicin at 15 mg/kg	51	36 (70.6)	56.2–82.5	9 (17.6)	6 (11.8)
	Levofloxacin at 750 mg	29	19 (65.5)	45.7–82.1	7 (24.1)	3 (10.3)
ME	Plazomicin at 10 mg/kg	7	4 (57.1)	18.4–90.1	3 (42.9)	–
	Plazomicin at 15 mg/kg	35	28 (80.0)	63.1–91.6	7 (20.0)	–
	Levofloxacin at 750 mg	21	16 (76.2)	52.8–91.8	5 (23.8)	–

a –, patients with missing or indeterminate outcome data were excluded from the ME population.

The clinical cure rates for patients with antibiotic-resistant Enterobacteriaceae at the baseline are shown in Table 5. Of the 19 patients in the MITT population with levofloxacin-resistant Enterobacteriaceae, clinical cure was achieved in 75.0% (3/4), 77.8% (7/9), and 66.7% (4/6) in the groups receiving plazomicin at 10 mg/kg and 15 mg/kg and levofloxacin, respectively. Favorable cure rates were also achieved with plazomicin for patients with Enterobacteriaceae that were nonsusceptible to amikacin or resistant to ceftazidime, gentamicin, or trimethoprim-sulfamethoxazole at the baseline (Table 5).

**TABLE 5:** Clinical cure at TOC for patients with antibiotic-resistant Enterobacteriaceae at baseline (MITT population)^*a*^

Enterobacteriaceae susceptibility	No. of patients with cure at TOC/no. of patients with the specified pathogen at baseline (%)		
	Plazomicin at 10 mg/kg	Plazomicin at 15 mg/kg	Levofloxacin at 750 mg
Levofloxacin resistant	3/4 (75.0)	7/9 (77.8)	4/6 (66.7)
Ceftazidime resistant	3/4 (75.0)	2/3 (66.7)	4/5 (80.0)
Gentamicin resistant	3/3 (100)	3/3 (100)	4/4 (100)
Amikacin nonsusceptible	0	1/1 (100)	1/1 (100)
TMP-SMX resistant	3/4 (75.0)	13/16 (81.3)	5/7 (71.4)

a Resistance and nonsusceptibility were defined according to CLSI and EUCAST breakpoints as follows: for levofloxacin, MIC > 4 μg/ml; for ceftazidime, CLSI MIC ≥ 16 μg/ml and EUCAST MIC > 4 μg/ml; for gentamicin, CLSI MIC ≥ 16 μg/ml; for amikacin, CLSI MIC ≥ 64 μg/ml and EUCAST MIC ≥ 16 μg/ml; and for trimethoprim-sulfamethoxazole, CLSI MIC ≥ 4 μg/ml and EUCAST MIC > 4 μg/ml (42, 43). TMP-SMX, trimethoprim-sulfamethoxazole.

### Safety.

AEs were reported in 31.8% (7/22), 35.1% (26/74), and 47.7% (21/44) of the patients in the groups receiving plazomicin at 10 mg/kg and 15 mg/kg and levofloxacin, respectively (Table 6). The most frequent AEs reported in the plazomicin treatment groups included headache, nausea, vomiting, diarrhea, and dizziness, and most were graded by the investigator to be mild or moderate in severity.

**TABLE 6:** Safety analysis (safety population)

Event^*a*^	Values for patients receiving:		
	Plazomicin at 10 mg/kg (*n* = 22)	Plazomicin at 15 mg/kg (*n* = 74)	Levofloxacin at 750 mg (*n* = 44)
No. (%) of patients with any AE	7 (31.8)	26 (35.1)	21 (47.7)
No. (%) of patients with the following AEs reported in ≥5% of patients in any treatment group:			
Headache	2 (9.1)	6 (8.1)	3 (6.8)
Diarrhea	0 (0.0)	4 (5.4)	2 (4.5)
Vomiting	0 (0.0)	4 (5.4)	1 (2.3)
Nausea	0 (0.0)	4 (5.4)	0 (0.0)
Dizziness	0 (0.0)	4 (5.4)	0 (0.0)
No. (%) of patients with:			
AE related to renal function^*b*^	0 (0.0)	2 (2.7)	0 (0.0)
AE related to vestibular or cochlear function^*c*^	0 (0.0)	2 (2.7)	1 (2.3)
AE related to study drug	2 (9.1)	15 (20.3)	12 (27.3)
AE leading to study drug discontinuation	0 (0.0)	4 (5.4)	1 (2.3)
Any serious AE	0 (0.0)	1 (1.4)	2 (4.5)
No. of patients with a ≥0.5-mg/dl increase in serum creatinine concn/total no. of patients tested (%):			
At any time during the study	1/22 (4.5)	4/72 (5.6)	1/41 (2.4)
While on i.v. study drug	0/22 (0.0)	3/72 (4.2)	0/41 (0.0)

a AEs were coded according to the preferred terms in version 12.1 of MedDRA.

b Events include preferred terms of azotemia and acute renal failure.

c Events include preferred terms of tinnitus and vertigo and worsening of audiometry.

Three patients experienced a serious AE during the study. One was a patient in the group receiving plazomicin at 15 mg/kg, who had a negative pregnancy test at enrollment but experienced a spontaneous abortion on day 103 that was not considered related to study drug. The other two were patients in the levofloxacin group: one with a convulsion considered related to study drug and one with recurrent AP resulting in nephrectomy considered unrelated to study drug. There were no deaths during the study.

Five patients (four in the group receiving plazomicin at 15 mg/kg and one in the group receiving levofloxacin) had AEs that led to discontinuation of study drug. A 24-year-old woman experienced severe hypotension (systolic/diastolic blood pressure, 80/50 mm Hg) along with moderate dizziness on day 3, 30 min after receiving both the 30-min plazomicin infusion and the 90-min placebo infusion. The hypotension resolved at 2 h 15 min after onset, at which time the patient's blood pressure was 100/70 mm Hg. Both AEs (hypotension and dizziness) were considered related to study drug and resolved without sequelae. A 55-year-old woman discontinued plazomicin due to a worsening of preexisting diabetes mellitus (not related to study drug) and mild azotemia on day 3 of dosing (considered related to study drug), when her serum creatinine level increased from a baseline value of 1.6 mg/dl to 2.1 mg/dl on the day of the AE. At LFU, the patient's serum creatinine level was 1.8 mg/dl. Two patients, one with moderate dizziness and one with mild transient vertigo, discontinued plazomicin after two doses; both were considered related to study drug and resolved without sequelae. One patient discontinued levofloxacin due to a serious AE of convulsion that was considered related to study drug.

AEs associated with renal function occurred in two patients in the group receiving plazomicin at 15 mg/kg (Table 6): the previously described patient with mild azotemia that led to drug discontinuation and one patient with mild acute renal insufficiency whose serum creatinine level increased from 0.9 mg/dl at the baseline to 1.6 mg/dl at TOC and returned to near the baseline level (1.1 mg/dl) at LFU. For most patients, serum creatinine levels remained stable throughout the study. Five plazomicin-treated patients and one levofloxacin-treated patient had a ≥0.5-mg/dl increase in the serum creatinine level from that at the baseline during the study, including three patients in the group receiving plazomicin at 15 mg who had an increase while on intravenous (i.v.) therapy. The levels returned to near baseline by LFU in all but one plazomicin-treated patient, whose serum creatinine level increased from a baseline value of 1.2 mg/dl (normal range, 0.7 to 1.3 mg/dl) to 2.0 mg/dl on day 5 at the end of treatment and remained elevated (1.8 mg/dl) at LFU.

No patient met Hy's law criteria for liver injury (45).

AEs possibly associated with vestibular and cochlear function occurred in two patients in the group receiving plazomicin at 15 mg/kg and one patient in the group receiving levofloxacin (Table 6). As described above, one patient experienced mild transient vertigo after receiving the second dose of plazomicin. Her vertigo resolved without sequelae, and modified Romberg and audiogram test results were normal. Another patient, a 47-year-old woman with normal audiogram results, experienced mild unilateral tinnitus 14 days after the end of plazomicin therapy. Repeat audiograms at LFU and 3 months after the end of treatment showed normal hearing levels; however, mild unilateral tinnitus persisted at 3 months. One patient in the levofloxacin group experienced mild worsening of audiometry results at TOC; a small but consistent drop in hearing levels was detected, ranging from 10 to 40 dB in the frequencies tested (250 to 8,000 Hz), but the results did not meet the predefined sponsor criteria for sensorineural abnormalities. Modified Romberg testing generally showed no clinically important changes in vestibular function over the study; of the 93 patients who completed this testing, 89 showed normal results both at the baseline and at study days 10 to 14, and 2 showed a mild decline from the results at the baseline (1 in the plazomicin treatment group and 1 in the levofloxacin treatment group). No plazomicin-treated patients with evaluable audiometry data (0/75) showed postbaseline sensorineural, conductive, or mixed hearing loss.

There were no clinically important trends in laboratory parameters, vital signs, or electrocardiograms for patients who received plazomicin. AEs associated with vital signs were reported for four patients: the previously mentioned patient with severe hypotension that led to discontinuation of plazomicin and three patients with mild or moderate hypertension considered unrelated to study drug (one in the group receiving plazomicin at 10 mg/kg and two in the group receiving levofloxacin).

## DISCUSSION

The results of this small phase 2 study demonstrate that the administration of plazomicin (10 or 15 mg/kg) once daily for 5 days was an effective treatment in adult patients with cUTI, including AP. Microbiological eradication was achieved in over 85% of plazomicin-treated patients in the ME population, and 80% of patients who received the 15-mg/kg dose of plazomicin were assessed by the investigator to be clinically cured, with complete resolution of baseline signs and symptoms of infection. Additionally, a lower rate of microbiological recurrence at LFU, which occurred ∼1 month after the last dose of study drug, was observed in the 15-mg/kg plazomicin treatment group than in the levofloxacin treatment group (6.5% versus 23.5%).

There are few large randomized, controlled trials that have evaluated the efficacy of aminoglycosides dosed once daily for cUTI or AP. Despite the small sample size of the current study and challenges in comparing data across studies with methodological differences, the high microbiological eradication rate observed with once-daily plazomicin therapy in the ME population was similar to that reported to be achieved with other aminoglycosides when dosed once daily (46, 47). Specifically, once-daily isepamicin therapy (8 or 15 mg/kg, depending on the severity of infection) was associated with microbiological eradication in 91% (92/101) of evaluable patients 4 to 15 days after the end of treatment (46), and once-daily amikacin therapy (15 mg/kg/day) was associated with microbiological eradication in 94% (32/34) of patients 7 to 10 days after the end of treatment (47). These numbers are comparable to the 89% (31/35) microbiological eradication rate at TOC for plazomicin (15 mg/kg) seen in the ME population in our study.

The clinical cure rates achieved with plazomicin were generally favorable in the small number of patients with antibiotic-resistant Enterobacteriaceae at the baseline, including aminoglycoside-resistant isolates (3/3 patients with gentamicin-resistant isolates and 1/1 patient with amikacin-resistant isolates in the 15-mg/kg plazomicin group). Although investigations in a larger group of patients with cUTIs due to resistant pathogens are needed to more precisely quantify the response rates to these pathogens, the cure rates associated with plazomicin in this study are promising, particularly in the current clinical environment in which options for the treatment of MDR pathogens are limited.

The causative uropathogens isolated in this study are typical and similar to those isolated in recent clinical trials of cUTIs (48, 49), although in our study relatively few P. mirabilis and P. aeruginosa isolates were recovered, perhaps due to the small number of patients enrolled. Levofloxacin resistance was detected in 28% of Enterobacteriaceae isolates recovered at the baseline in this study, which completed enrollment in 2012. Similarly high rates of levofloxacin resistance have been detected in recent registrational trials for this indication (48, 49), potentially introducing bias into noninferiority trials of cUTI and leading several experts to suggest that levofloxacin is no longer an appropriate choice of comparator for a noninferiority study or for empirical therapy of cUTI in patients with risk factors for antibiotic resistance (50, 51). In the context of this study, relatively few levofloxacin-resistant (MIC > 4 μg/ml) uropathogens were included in the primary analysis populations of the levofloxacin treatment group (6/25 and 3/19 in the MITT and ME populations, respectively), suggesting that study results may not have been overly impacted by fluoroquinolone resistance.

In this study, we closely monitored patients for toxicities associated with aminoglycosides, including nephrotoxicity and ototoxicity, although once-daily dosing has been associated with lower rates of nephrotoxicity than traditional dosing (23, 26, 29). Plazomicin administered at 10 or 15 mg/kg once daily as a 30-min infusion for 5 days was generally well tolerated by patients with cUTI or AP. The incidence of AEs was similar between the levofloxacin and plazomicin treatment groups, and most of the AEs were mild to moderate in intensity and rapidly resolved. In most patients, serum creatinine levels remained stable over the study. Postbaseline serum creatinine level increases of ≥0.5 mg/dl, which are considered to be a clinically meaningful measure of new-onset renal dysfunction (52), were observed in five plazomicin-treated patients and one levofloxacin-treated patient. These events resolved in all but one plazomicin-treated patient. These safety results are consistent with the known mechanism of aminoglycoside toxicity and phase 1 study data for healthy adults who received a once-daily plazomicin regimen of 15 mg/kg administered for 5 days (53). Additionally, no treatment-related changes in audiometry were observed.

Our study is limited by the small sample size, the limited number of patients with infections due to resistant pathogens, and the relatively large percentage of patients with an indeterminate outcome in the MITT population in each treatment group. Indeterminate outcomes at TOC were due to urine culture samples that were either contaminated, not obtained, or obtained outside the permitted window of 5 to 12 days after the end of treatment. Another limitation was the underrepresentation of patients from Europe, as patients from that continent were not enrolled. However, plazomicin activity and exposures are not expected to be impacted by race or ethnicity.

In summary, the results of this small phase 2 study suggest that plazomicin dosed at 15 mg/kg once daily for 5 days is effective in the treatment of adults with cUTIs, including patients with antibiotic-resistant Enterobacteriaceae. This dose and duration of plazomicin were well tolerated overall, with mild and generally reversible increases in serum creatinine levels being noted in a small number of patients. Although our findings suggest a potential dose-response effect of plazomicin on serum creatinine levels, the numbers are too small to draw conclusions and larger studies will be required to define exposure-response relationships for nephrotoxicity. Plazomicin has the potential to address an unmet medical need for patients with cUTIs or AP caused by MDR Enterobacteriaceae, and further studies using once-daily 15-mg/kg dosing are warranted.

## MATERIALS AND METHODS

### Study design.

This multicenter, double-blind, randomized, comparator-controlled, phase 2 study was conducted from 13 July 2010 to 3 April 2012 at 27 study sites in the United States, India, Colombia, and Chile. This study is registered at ClinicalTrials.gov under the identifier NCT01096849 and was performed in accordance with the Declaration of Helsinki, the International Conference on Harmonisation/Good Clinical Practice guidelines, and applicable regulatory requirements. An independent ethics committee or institutional review board at each site approved the protocol, and patients were required to provide written informed consent.

### Inclusion and exclusion criteria.

Inpatients and outpatients were eligible for study participation. Patients participating on an outpatient basis were to receive study drug infusions and all other study activities by home health care or have the ability to return to the study center. Eligibility criteria included the following: age, 18 to 85 years; body weight, ≤100 kg, and a documented or suspected cUTI or AP with protocol-specified clinical signs and symptoms. A cUTI was defined by the presence of pyuria (≥5 white blood cells [WBC] per high-power field in urine sediment and/or a positive leukocyte esterase test on urinalysis) and at least one of the following signs or symptoms: fever (oral temperature, ≥38.5°C or ≥101.3°F), elevated WBC count (≥10,000/mm^3^ or a left shift of ≥15% immature polymorphonuclear leukocytes), dysuria, increased urinary frequency, urgency, or lower abdominal pain. At least one of the following complicating factors was also required in the definition of a cUTI: an indwelling catheter (to be removed or replaced by ≤12 h after randomization), urine residual volume of ≥100 ml, neurogenic bladder, or urinary retention in men due to previously diagnosed benign prostatic hypertrophy. AP was defined as the presence of signs or symptoms of an ascending tract infection, including fever or an elevated WBC count, lower back/flank pain, pyuria, and at least one of the following: costovertebral angle tenderness, nausea, chills, dysuria, increased urinary frequency, urgency, or vomiting. All patients were required to have a creatinine clearance (CL_CR_) of ≥60 ml/min using the Cockcroft-Gault formula (54).

Patients were excluded from the study if they had acute bacterial prostatitis, orchitis, epididymitis, chronic bacterial prostatitis, gross hematuria requiring intervention other than study drug, urinary tract surgery within 7 days before randomization or planned during the study period, a known nonrenal source of infection diagnosed within 7 days of randomization, a QTc interval of >440 ms, a history of hearing loss before the age of 40 years, sensorineural hearing loss, or a family history of hearing loss. Pregnant or breast-feeding women were also excluded. Patients were not permitted to receive any systemic antibacterial (oral, i.v., intramuscular) therapy or antibacterial bladder irrigation for the treatment of a bacterial cUTI or AP in the 48 h preceding randomization or during the study. Full inclusion and exclusion criteria are provided in the supplemental material.

### Randomization and treatment.

Patients were enrolled and randomized (1:1:1) by a central interactive voice response system to receive i.v. plazomicin (10 or 15 mg/kg) or i.v. levofloxacin (750 mg) once daily for 5 days. Enrollment in the 10-mg/kg treatment group was stopped during the study to allow preferential enrollment in the higher-dose group (15 mg/kg). Patients were subsequently randomized in a 2:1 ratio to receive i.v. plazomicin (15 mg/kg) or i.v. levofloxacin (750 mg). Randomization was stratified by type of infection (AP, cUTI with an indwelling catheter, or cUTI without an indwelling catheter). Patients and investigators were blind to the treatment assignment. To maintain the blind, patients received two i.v. infusions on each of the 5 treatment days: a 30-min plazomicin infusion followed by a 90-min placebo infusion or a 30-min placebo infusion followed by a 90-min levofloxacin infusion. The plazomicin regimens were selected on the basis of the pharmacokinetic profile and the safety data observed in two previously completed phase 1 studies (53). The high-dose, short-course levofloxacin regimen used in this study is widely considered the standard of care for cUTI (3, 24, 55) and is approved as a 5-day regimen.

### Analysis populations.

The intent-to-treat population included all randomized patients. The safety population included all randomized patients who received any amount of study drug. The MITT population included all randomized patients with at least one isolated causative bacterial pathogen at ≥10^5^ CFU/ml from an appropriately collected pretreatment urine specimen. Patients with isolates present at ≥10^4^ CFU/ml and <10^5^ CFU/ml were evaluated for inclusion in conjunction with urinalysis and clinical signs and symptoms data. Patients were considered clinically evaluable if they had a protocol-defined cUTI or AP, received study medication as randomized, were blind to treatment assignment (except in the event of a treatment-emergent AE or serious AE), and received ≥80% of study drug for clinical success or 40% for clinical failure and the clinical response was assessed at TOC. The ME population included all clinically evaluable patients with a causative pathogen isolated at the baseline and results obtained from a noncontaminated urine culture collected at TOC within a specified window (5 to 12 days after the end of treatment, except in cases of early failure, in which the TOC visit was performed early). Patients with missing or indeterminate outcome data were excluded from the ME population.

### Efficacy endpoints and assessments.

The coprimary efficacy endpoints were microbiological eradication in the MITT and the ME populations at TOC, 5 to 12 days after the last treatment. Secondary endpoints included microbiological eradication at TOC by primary diagnosis (cUTI or AP) and baseline uropathogen, the investigator's assessment of clinical cure at TOC, the time to clinical cure, and microbiological recurrence and clinical relapse at LFU, 33 to 47 days after the last treatment. Clinical outcomes in patients with antibiotic-resistant pathogens at the baseline were also assessed.

Microbiological outcomes were defined as eradication (TOC urine culture with <10^4^ CFU/ml of the baseline pathogen), noneradication (TOC urine culture with ≥10^4^ CFU/ml of the baseline pathogen), and indeterminate (any uropathogen that could not be classified as eradicated or persistent [including missing data]). Microbiological recurrence was defined as an LFU urine culture with >10^5^ CFU/ml of regrowth of a baseline pathogen that was eradicated at TOC. Clinical outcomes were defined as cure (complete resolution of all baseline signs and symptoms at TOC without the use of additional antibiotic therapy or an AE leading to premature discontinuation of the study drug), failure (persistence, incomplete resolution, or worsening of baseline clinical signs and symptoms or development of new clinical signs and symptoms of cUTI or AP requiring additional antimicrobial therapy at any time through the TOC visit), and indeterminate (an outcome other than cure or failure, including loss to follow-up before the TOC visit). Clinical relapse at LFU was defined as the return of clinical signs and symptoms requiring antibiotic therapy in patients who were clinically cured at TOC.

Baseline pathogens were sent to a central laboratory (Eurofins Global Central Laboratory, Chantilly, VA) for identification and susceptibility testing. Results were interpreted according to CLSI (42) and EUCAST (43) criteria. The MICs of plazomicin, levofloxacin, and other comparator antibiotics, including amikacin, gentamicin, ceftazidime, and trimethoprim-sulfamethoxazole, were determined using broth microdilution. Enterobacteriaceae species were classified as having an MDR phenotype if they were nonsusceptible to at least one agent in three or more antimicrobial categories (44).

### Safety assessments.

Safety investigations included physical examinations, measurement of vital signs, electrocardiograms, collection of reports of AEs, laboratory tests, cochlear tests (pure tone audiometry [PTA] with bone conduction at frequencies of up to 20,000 Hz with a minimum upper limit of 8,000 Hz), and vestibular function (modified Romberg) tests. AE reports, vital sign measurements, and blood and urine for safety analyses were collected at screening, daily while on treatment, and at TOC and LFU. Laboratory safety tests (hematology, serum chemistry, and urinalysis) were performed centrally at the ACM Medical Laboratory (Rochester, NY). An independent licensed audiologist reviewed the PTA with bone conduction results to assess changes from the baseline according to predefined criteria. Sensorineural hearing loss was defined as abnormal pure tone air and bone conduction of ≥25 dB and an air-bone gap of ≤10 dB, conductive hearing loss was defined as a normal bone conduction (<25 dB) and air conduction higher (worse) than bone conduction by >10 dB, and mixed hearing loss was defined as abnormal pure tone air and bone conduction (≥25 dB). Modified Romberg testing evaluated neurological function (including proprioception), vestibular function, and vision across four test conditions; a classification of “normal” required passing all four test conditions with no symptoms of disequilibrium or vertigo (56).

### Statistical analyses.

This study was not powered for inferential statistics. With assumed microbiological eradication rates of 85% and 88% in the plazomicin treatment group in the MITT and ME populations, respectively, the final overall sample size of 145 patients (98 in the plazomicin treatment group) provided for 95% CIs of 73 to 94% and 74 to 95%, respectively.

Two-sided 95% CIs were calculated for the point estimates of microbiological eradication within each treatment group (Clopper-Pearson method) and for the difference in eradication rates between the plazomicin and levofloxacin treatment groups (based on the normal approximation with a continuity correction). Secondary efficacy endpoints were analyzed using descriptive statistics and CIs, where appropriate. Time-to-event analyses were conducted using Kaplan-Meier methods. Safety data were summarized descriptively.

## Supplementary Material

Supplemental material
