# Supplementary material for: A Multicenter, Randomized, Double-Blind, Phase 2 Study of the Efficacy and Safety of Plazomicin Compared with Levofloxacin in the Treatment of Complicated Urinary Tract Infection and Acute Pyelonephritis
Source: Antimicrob Agents Chemother. 2018 Mar 27;62(4):e01989-17. doi: 10.1128/AAC.01989-17 (PMC5913993; doi:10.1128/AAC.01989-17)
Supplement: Supplemental material [file supp_62_4_e01989-17__index.html]

A Multicenter, Randomized, Double-Blind, Phase 2 Study of the Efficacy and Safety of Plazomicin Compared with Levofloxacin in the Treatment of Complicated Urinary Tract Infection and Acute Pyelonephritis — Supplemental material 

# A Multicenter, Randomized, Double-Blind, Phase 2 Study of the Efficacy and Safety of Plazomicin Compared with Levofloxacin in the Treatment of Complicated Urinary Tract Infection and Acute Pyelonephritis

## Supplemental material

- Supplemental file 1 -

  Supplemental text

  PDF, 256K
