# Supplementary material for: A Multicenter, Randomized, Double-Blind, Phase 2 Study of the Efficacy and Safety of Plazomicin Compared with Levofloxacin in the Treatment of Complicated Urinary Tract Infection and Acute Pyelonephritis
Source: Antimicrob Agents Chemother. 2018 Mar 27;62(4):e01989-17. doi: 10.1128/AAC.01989-17 (PMC5913993; doi:10.1128/AAC.01989-17)
Supplement: Supplemental material [file AAC.01989-17_zac004187030s1.pdf]

SUPPLEMENTARY APPENDIX

Inclusion criteria. Patients who met all of the following inclusion criteria were eligible to participate in the study:

1. Able to understand and provide written informed consent. (If the patient was unable to sign for reasons other than cognitive, a legally authorized representative could sign.)

2. Age ≥18 and ≤85 years.

3. An estimated dry body weight (nonedematous or non–volume-expanded weight) of ≤100 kg.

4. Documented or suspected complicated urinary tract infection (cUTI) or acute pyelonephritis (AP) with clinical signs and symptoms as specified below (documented cUTI or AP was defined by a positive prerandomization urine culture with a defined urinary bacterial pathogen):

- Presence of cUTI, manifested by pyuria, defined as ≥5 white blood cells (WBCs)/high-power field (hpf) in urine sediment and/or a positive leukocyte esterase (LCE) test on urinalysis AND at least ONE of the following:
    - Fever (oral temperature of ≥38.5°C or ≥101.3°F), or elevated WBC (≥10,000/mm^3 or left shift ≥15% immature polymorphonuclear leukocytes [PMNs])
    - Dysuria
    - Increased frequency
    - Urgency
    - Lower abdominal pain AND at least ONE of the following:
        - Indwelling catheter
        - Urine residual volume ≥100 ml (within the past 6 months) as previously determined by voiding cystourethrogram, or ultrasound, or straight catheterization immediately post void
        - Neurogenic bladder (excludes dropped bladders without documented residual volume)
        - Urinary retention in men due to previously diagnosed benign prostatic hypertrophy

OR:

- Presence of clinical signs and symptoms of an ascending tract infection (AP), manifested by:
    - Fever (oral temperature of ≥38.5°C or ≥101.3°F) OR elevated WBC (≥10,000/mm^3 or left shift ≥15% immature PMNs)

- Lower back/flank pain
- Pyuria defined as ≥5 WBCs/hpf in urine sediment and/or a positive LCE on urinalysis AND at least ONE of the following:
    - Costovertebral angle tenderness
    - Nausea
    - Chills
    - Dysuria
    - Increased urinary frequency
    - Urgency
    - Vomiting.

5. Expectation that patients with indwelling catheters would have the catheter removed or replaced (if removal was not clinically acceptable) before or as soon as possible, but not longer than 12 h after randomization.

6. Normal renal function at screening as estimated by creatinine clearance ≥60 ml/min using the Cockcroft–Gault formula.

7. Women of childbearing potential: negative pregnancy test before randomization, not breastfeeding, and using a highly effective method of contraception for ≥1 month before randomization and through the completion of the study. A highly effective method of contraception for female patients included one of the following: hormonal implants/patch; injectable hormones; oral hormonal contraceptives; prior bilateral oophorectomy; prior hysterectomy; prior bilateral tubal ligation; intrauterine device; approved cervical ring; condom; true abstinence, if approved by the principal investigator; or a vasectomized partner.

8. Expectation that, in the judgment of the investigator, the patient would survive with effective antibiotic therapy and appropriate supportive care for the anticipated duration of the study.

9. Willing to comply with all study activities and procedures, whether in the hospital or after discharge, throughout the duration of the study.

Exclusion criteria. Patients who met any of the following criteria were not eligible to participate in the study:

1. Any of the following:
    - Perinephric abscess
    - Renal corticomedullary abscess
    - Uncomplicated urinary tract infection
    - Polycystic kidney disease
    - Only one functional kidney

- Obstructive uropathy, where the obstruction is unlikely to resolve or be relieved by stent or nephrostomy tube ≤24 h after randomization
- Chronic vesicoureteral reflux
- Previous or planned renal transplantation
- Previous or planned cystectomy or ileal loop surgery
- Known candiduria.

2. Presence of acute bacterial prostatitis, orchitis, epididymitis, or chronic bacterial prostatitis as determined by history and/or physical exam.

3. Gross hematuria requiring intervention other than study drug.

4. Urinary tract surgery within 7 days before randomization or urinary tract surgery planned during the study period (except surgery required to relieve an obstruction or place a stent or nephrostomy).

5. Known nonrenal (excluding bacteremia) source of infection such as endocarditis, osteomyelitis, abscess, meningitis, or pneumonia diagnosed within 7 days before randomization.

6. Any of the following signs of severe sepsis:
   - Shock or profound hypotension, defined as systolic blood pressure <90 mm Hg or a decrease of >40 mm Hg from baseline that is not responsive to fluid challenge
   - Hypothermia (core temperature <35.6°C or <96.1°F)
   - Disseminated intravascular coagulation as evidenced by prothrombin time, partial thromboplastin time 2× the upper limit of normal, or platelets <50% of the lower limit of normal.

7. Pregnant or breastfeeding women.

8. Myasthenia gravis or other neuromuscular disorder.

9. History of epilepsy or known seizure disorder (excluding a history of childhood febrile seizures).

10. Receipt of disulfiram during the 30 days preceding date of randomization and throughout the duration of the study.

11. Receipt of any investigational medicine during the past month (30 days or five half-lives, whichever is longer) before randomization.

12. Prior exposure to plazomicin.

13. Treatment with another antibiotic within 48 h before randomization.

14. Treatment with a fluoroquinolone systemically administered (i.e., intravenous or oral) or administered as a bladder irrigation within 30 days before randomization if, in the judgment of the investigator, this would have exposed the patient to unnecessary risk.

15. Known history of HIV infection and current or previous CD4 count <200/mm^3.

16. Presence of immunodeficiency or an immunocompromised condition including hematologic malignancy, bone marrow transplant, or receiving immunosuppressive therapy such as cancer chemotherapy, medications for the rejection of transplantation, and long-term (≥2 weeks) use of systemic corticosteroids.

17. Presence of neutropenia (<1,000 PMNs/mm^3).

18. History of hearing loss with onset before age 40 years, sensorineural hearing loss, or a family history of hearing loss (excluding age-related hearing loss [onset after age of 65 years]).

19. At risk for serious drug interactions because of concomitant medications (class IA [e.g., quinidine, procainamide] or class III [e.g., amiodarone, sotalol] antiarrhythmic agents).

20. A QTc (Bazett's) >440 ms.

21. Any disorder that could interfere with the evaluation of study drug.

22. Known hypersensitivity to aminoglycosides, levofloxacin, other fluoroquinolone antibiotics, or any of the excipients used in the study drug formulations.

23. Unable or unwilling, in the judgment of the investigator, to comply with the protocol.

24. An employee of the investigator or study center with direct involvement in the proposed study or other studies under the direction of that investigator or study center, as well as a family member of the employee or the investigator.
